# CREB3L1 as a potential biomarker predicting response of triple negative breast cancer to doxorubicin-based chemotherapy

**DOI:** 10.1186/s12885-018-4724-8

**Published:** 2018-08-13

**Authors:** Bray Denard, Sharon Jiang, Yan Peng, Jin Ye

**Affiliations:** 1 Department of Molecular Genetics, University of Texas Southwestern Medical Center, 5323 Harry Hines Blvd., Dallas, TX 75390-9046 USA; 2 Department of Pharmacology, University of Texas Southwestern Medical Center, 5323 Harry Hines Blvd., Dallas, TX 75390-9041 USA; 3 Department of Pathology, University of Texas Southwestern Medical Center, 5323 Harry Hines Blvd., Dallas, TX 75390-9073 USA

**Keywords:** Triple negative breast cancer, doxorubicin, chemotherapy, CREB3L1

## Abstract

**Background:**

Doxorubicin-based chemotherapy is currently the most frequently used treatment for triple negative breast cancer (TNBC), yet the response rate is not high due to the lack of a biomarker allowing identification of responsive patients before the chemotherapy is initiated. We have demonstrated that doxorubicin inhibits proliferation of cancer cells through proteolytic activation of a transcription factor called CREB3L1 (cAMP response element binding protein 3-like 1), and that CREB3L1 expression in cancer cells is a key determinant of their sensitivity to doxorubicin when they are cultured *in vitro* or established as xenograft tumors in mice. The purpose of this study is to determine whether CREB3L1 expression in tumor cells of TNBC patients can be established as a biomarker to predict outcomes of doxorubicin-based chemotherapy.

**Methods:**

We performed a retrospective analysis on breast core biopsy tissue samples taken from 18 TNBC patients before they were treated with doxorubicin-based chemotherapy. CREB3L1 expression in the cancer cells was analyzed by immunohistochemistry and quantified using the Immunoreactive Score (IRS). Outcomes of the chemotherapy were measured by the residual cancer burden (RCB) system.

**Results:**

CREB3L1 expression levels in TNBC responsive to doxorubicin-based chemotherapy (RCB class 0-2) were significantly higher than that in resistant cancers (RCB class 3) (unpaired two-tailed t test, *p* = 0.0005; Statistical power 99.8 at 95% confidence level). All cancers expressing higher levels of CREB3L1 (IRS 4-12) responded to doxorubicin-based chemotherapy, whereas all cancers resisting the treatment expressed lower levels of CREB3L1 (IRS 0-3).

**Conclusions:**

These results suggest that CREB3L1 expression level may be used as a biomarker to identify TNBC patients who are more likely to benefit from doxorubicin-based chemotherapy.

## Background

Breast cancer can be divided into subgroups based on expression of estrogen receptor, progesterone receptor, and human epidermal growth factor receptor 2, which are growth factor receptors that are crucial for proliferation of cancer cells. While targeted therapies antagonizing these receptors have significantly improved the treatment of breast cancers expressing these receptors, treating those that express none of the three receptors, namely triple negative breast cancer (TNBC), remains a daunting challenge [1]. Chemotherapy is still the mainstream treatment for these cancers. Nearly all chemotherapy regimens used to treat TNBC contain multiple drugs, and the majority of those include doxorubicin due to its effectiveness demonstrated through decades of clinical practice [2]. However, the response rate of doxorubicin-based chemotherapy is only 40–70%, primarily because there is no biomarker capable of identifying patients who will respond to doxorubicin [3–5]. Considering the severe cardiac toxicity caused by the side effect of doxorubicin [6], establishment of such a biomarker will not only improve the response rate of doxorubicin-based chemotherapy, but also reduce the toxicity of the treatment by avoiding application of doxorubicin to patients who are unlikely to respond to doxorubicin.

Doxorubicin has been demonstrated to induce DNA damage by inhibiting topoisomerase II, but whether this activity is associated with its ability to suppress cancer cell proliferation is still under debate [7, 8]. We have recently reported that doxorubicin blocks proliferation of cancer cells through activation of a transcription factor called cAMP response element binding protein 3-like 1 (CREB3L1) [9]. Unlike most transcription factors, CREB3L1 is synthesized as an inactive transmembrane precursor, with the transcriptionally active N-terminal domain anchored to membranes via a single transmembrane helix [10]. Doxorubicin stimulates proteolytic cleavage of CREB3L1, releasing the N-terminal domain of the protein from membranes, allowing it to enter nucleus where it activates transcription of genes that inhibit cell proliferation [9, 10]. As a result, doxorubicin inhibited proliferation of tumor cells cultured *in vitro* that express CREB3L1 but not those in which expression of the gene was inhibited, even though DNA damage induced by the drug was indistinguishable among these cells [9]. Using a mouse xenograft model of human renal cell carcinoma reported to maintain drug sensitivities displayed in patients [11], we previously reported that doxorubicin at a dose lower than that typically applied to human patients shrank tumors expressing high levels of CREB3L1 but not those expressing low levels of the protein [12]. Notably, at this low dose doxorubicin did not induce DNA damage in the xenograft tumors regardless of their CREB3L1 expression levels [12]. These *in vitro* and *in vivo* results suggest that doxorubicin inhibits tumor cell proliferation through proteolytic activation of CREB3L1 but not DNA damage. These findings demonstrate that CREB3L1 expression in cancer cells could be a key determinant for their sensitivity to doxorubicin.

In the current study, we performed a retrospective analysis on core biopsy samples taken from TNBC patients before chemotherapy to measure CREB3L1 expression levels in cancer cells, and to determine the relationship between CREB3L1 expression levels and outcomes of doxorubicin-based chemotherapy. The results showed that CREB3L1 expression levels in cancers that responded to chemotherapy were significantly higher than those that resisted the treatment. Our findings suggest that high levels of CREB3L1 expression in cancer cells could serve as a predictive biomarker to identify TNBC patients who are likely to respond to doxorubicin-based chemotherapy.

## Methods

### Materials

We obtained a rabbit polyclonal antibody against CREB3L1 from Proteintech (Cat# 11235-2-AP); peroxidase-conjugated secondary antibodies from Jackson ImmunoResearch; and rabbit anti-Actin from Sigma-Aldrich. A mouse monoclonal antibody against human CREB3L1 (10H1) was generated by immunizing mice with synthesized polypeptides corresponding to amino acids 7-41 of human CREB3L1 [12]. Formalin fixed and paraffin embedded tissue sections of TNBC core biopsies were obtained from UT Southwestern University Hospitals. Written informed consent for participation in the study was obtained from participants or, where participants were children, a parent or guardian. All animal experiments reported previously that were mentioned in the manuscript were approved by an institutional animal care and use committee of UT Southwestern Medical Center (APN 2011-0192). No animal experiment is conducted in the current study.

### Verification of antibodies

Human hepatoma Huh7 cells stably transfected with a control shRNA or that targeting CREB3L1 were cultured as described previously [9]. Cells were lysed in buffer A (25 mM Tris-HCl pH = 7.2, 150 mM NaCl, and 1% NP-40) supplemented with cOmplete ULTRA protease inhibitor tablets (Roche) according to manufactures’ direction, analyzed by SDS-PAGE (10% acrylamide) followed by immunoblot analysis with the indicated antibodies (1:4000 dilution for 10H1, 1:1000 dilution for Proteintech 11235-2-AP, and 1:10,000 dilution for anti-Actin). Bound antibodies were visualized with a peroxidase-conjugated secondary antibody using the SuperSignal ECL-HRP substrate system (Pierce).

### Detection of CREB3L1 expression by Immunohistochemistry (IHC)

Eighteen TNBC core biopsy slides were subjected to IHC analysis with anti-CREB3L1 (10H1) to determine CREB3L1 expression in the tumors. Briefly, paraffin-embedded sections were treated with xylenes, washed sequentially with 95, 70, 50, 30% ethanol followed by a pure water wash. Endogenous peroxidase activity was blocked by incubating the slides with 3% hydrogen peroxide for 5 minutes. After washing with PBS, antigen retrieval was performed using the Retriever 2100 with Buffer U (Electron Microscopy Sciences) according to the manufactures’ directions. Sections were then blocked with Blocking Buffer (1% Horse Serum in PBS) for 1 hour, incubated with 17 μg/ml 10H1 overnight in Blocking Buffer at 4°C, washed three times with PBS, and developed with the DAB staining procedure using the Vectastain Elite ABC Kit and DAB Peroxidase (HRP) Substrate Kit (Vector Laboratories). Nuclei counterstaining was performed by incubating the slides with hematoxylin for 5 sec. The IHC results were captured by bright field images taken by a Zeiss Observer Z1 microscope using AxioVision software, and quantified through ImmunoReactive Score (IRS) as previously described [13]. The IRS quantification was verified by Dr. Yan Peng, a board-certified pathologist.

### Clinical characterization of tumors

Patient responses to doxorubicin-based chemotherapy applied after core biopsies had been taken were quantified through the residual cancer burden (RCB) system as previously described [14]. Expression of Ki67 in the tumor cells was quantified as previously reported [15].

### Statistical Analysis

We retrospectively analyzed TNBC patients whose core biopsies taken before doxorubicin-based chemotherapy were available, and whose response to the chemotherapy was recorded. We analyzed 18 samples meeting the requirement. A student t-test (two tailed, two sample unequal variance) was performed on results shown in Fig. 3a. Power calculation was performed on results shown in Fig. 3a at 95% confidence interval using a one-tail test. Pearson analysis was performed on results shown in Fig. 3b.

## Results

**Fig. 1:**
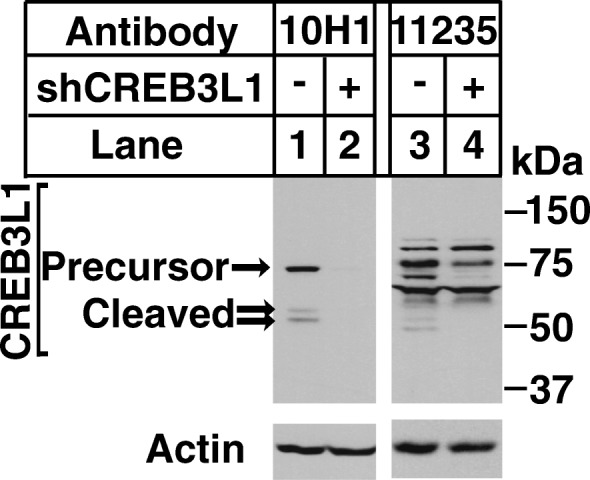
10H1 is a highly specific antibody against CREB3L1. On day 0, Huh7 cells stably transfected with a control shRNA or that targeting CREB3L1 were seeded at 4×10^5^ cells per 60 mm dish. On day 1, cells were harvest, and cell lysates were subjected to immunoblot analysis with antibodies reacting against CREB3L1 (10H1 or Proteintech Cat# 11235-2-AP) and Actin

A specific antibody against CREB3L1 without significant cross reactivity is required for IHC analysis of CREB3L1. The monoclonal antibody 10H1 we raised previously [12] is suitable for this application: On immunoblot the antibody detected only the precursor and cleaved form of CREB3L1 (Fig. 1, lane 1) in Huh7 cells stably transfected with a control shRNA that expressed CREB3L1, but detected no protein in Huh7 cells stably transfected with a shRNA targeting CREB3L1 that inhibited expression of the mRNA by more than 90% [9] (Fig. 1, lane 2). In contrast to 10H1, a commercially available anti-CREB3L1 stated suitable for IHC had much more cross reactivity (Fig. 1, lanes 3 and 4).

**Fig. 2:**
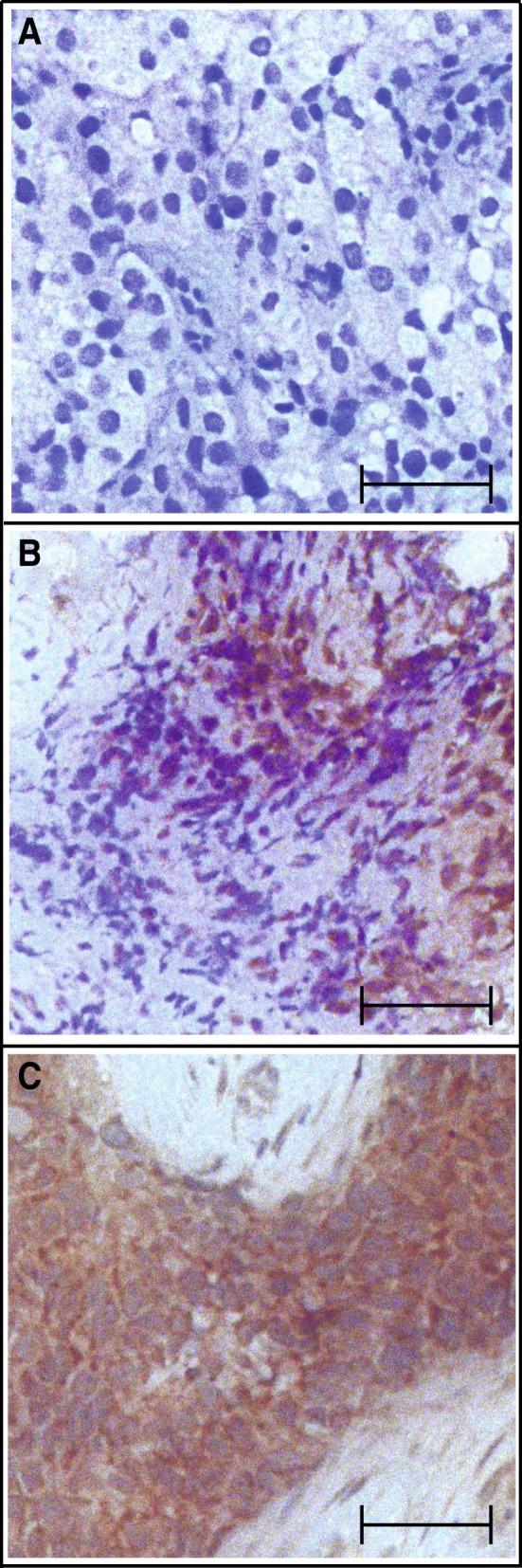
Representative CREB3L1 IHC results in TNBC core biopsies. IHC staining with 10H1 (brown) and hematoxylin (blue) was shown for case 12 (IRS value =0) (**a**), case 7 (IRS value =6) (**b**), and case 18 (IRS value = 8) (**c**) Scale bar: 50 μm

We then obtained unstained slides of core biopsies taken from TNBC patients before they were subjected to doxorubicin-based chemotherapy, and used 10H1 to measure CREB3L1 expression in the tumor cells through IHC. Some typical results of the IHC analysis were shown in Fig. 2, which include TNBC with no detectable expression (Fig. 2a), with heterogeneous expression (Fig. 2b), or with homogenous high levels of expression of CREB3L1 (Fig. 2c).

**Fig. 3:**
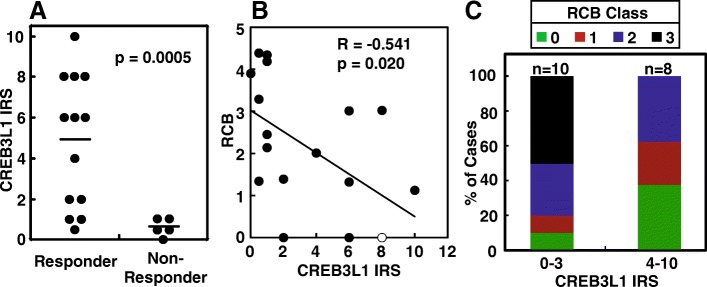
Higher levels of CREB3L1 expression in TNBC demonstrate an improved response to doxorubicin-based chemotherapy. **a** A student t-test (two tailed, two sample unequal variance) was performed to compare CREB3L1 IRS values in tumors responded to doxorubicin-based chemotherapy (RCB Class 0-2, responder) versus those resisted the treatment (RCB Class 3, non-responder). **b** Pearson analysis was performed to determine the correlation between CREB3L1 IRS values and RCB scores of the tumors. The hollow dot indicates two samples at the same location on the plot. **c** Percentage of cases with indicated RCB class in tumors with lower expression of CREB3L1 (IRS value 0-3) and those with higher expression of the protein (IRS value 4-12)

We quantified CREB3L1 expression in cancer cells of all patients before doxorubicin-based chemotherapy through the IRS system, and measured their response to the following chemotherapy through the RCB system. In the RCB system, the higher the score, the more resistant the tumor to chemotherapy [14]. Based on the RCB scores, the tumors are divided into 4 classes, with class 0, 1-2, and 3 refers to completely responsive, partially responsive, and completely resistant tumors, respectively [14]. The results were summarized in Table 1. According to our hypothesis, higher levels of CREB3L1 expression should render cancer cells more sensitive to doxorubicin. Consistent with this hypothesis, we observed that the average IRS value of tumors that completely or partially responded to the chemotherapy (RCB classes 0-2) was 8 times higher than that of the tumors resistant to the treatment (RCB class 3) (4.8 vs 0.6) (Fig. 3a). This difference is statistically significant indicated by the student t-test (*p* = 0.0005) and power analysis (statistical power 99.8% assuming a 95% confidence interval). Likewise, the degree of resistance to the chemotherapy measured by increased RCB scores was negatively correlated with CREB3L1 expression levels (Pearson analysis, *R* = -0.54, *p* = 0.02) (Fig. 3b).

**Table 1:** Patient information and data involved in the study

Case	Age	Chemotherapy in Addition to Doxorubicin	Pathologic TNM	Stage	Ki67	RCB Score	RCB Class	CREB3L1 IRS
1	40-49	Cytoxan, Taxol, Xeloda	ypT2pN3M1	IV	84	4.186	3	1
2	50-59	Cytoxan, Xeloda, Taxol	ypT2pN1M1	IV	45	4.387	3	0.5
3	50-59	Cytoxan, Taxol	ypT1bN0M0	IA	90	1.340	1	0.5
4	30-39	Cytoxan, Taxol	ypT2N3M0	IIIC	70	4.340	3	1
5	40-49	Cytoxan, Taxol	ypT0N1miM0	IB	95	1.125	1	10
6	50-59	Halaven, Paraplatin, Taxol	ypT2N1M1	IV	90	3.290	3	0.5
7	50-59	Cytoxan, Paraplatin, Taxol	ypT1cN1M0	IIA	30	3.014	2	6
8	40-49	Cytoxan, Taxol	ypT0N0M0	0	95	0	0	8
9	40-49	Cytoxan, Paraplatin, Taxol, Xeloda	ypT2N1M1	IV	35	1.390	2	2
10	40-49	Cytoxan, Gemzar, Paraplatin, Taxol	ypT1bN1M0	IIA	20	3.023	2	8
11	40-49	Cytoxan, Taxol	ypT2N0M0	0	60	0	0	2
12^a^	50-59	Arimidex, Cytoxan, Paraplatin, Taxol	ypT2N1M0	IIB	80	3.900	3	0
13	40-49	Cytoxan, Taxol, Xeloda	ypT2N0M0	0	85	2.452	2	1
14	70-79	Cytoxan, Taxol	ypT0N0M0	0	90	0	0	6
15	50-59	Cytoxan, Taxol	ypT1miN0M0	0	95	1.322	1	6
16^a^	50-59	Arimidex, Cytoxan, Taxol	ypT1cN0M0	IA	95	2.009	2	4
17^a^	40-49	Arimidex, Cytoxan, Taxol	ypT1cN0M0	IA	70	2.140	2	1
18^a^	60-69	Arimidex, Cytoxan, Taxol	ypTisN0M0	0	80	0	0	8

All patients were diagnosed and treated between 2011 and 2017 at UT Southwestern University Hospitals. Patient age range and chemotherapeutic drugs in addition to doxorubicin used in the treatment are shown. Response of the tumors to the chemotherapy measured through RCB Scores and RCB Classes, CREB3L1 expression in the tumors measured by IRS values, percentage of tumor cells positively stained by anti-Ki67, and other clinical characterization of the tumors is also presented

^a^These patients had two different breast cancers: one breast with TNBC; the other with ER positive cancer. Only TNBC but not the ER-positive tumors were analyzed in this study

To determine whether CREB3L1 expression levels can serve as a biomarker to predict outcomes of doxorubicin-based chemotherapy, we separated the tumors into two groups: One with lower (IRS ranges from 0 to 3) and the other with higher (IRS ranges from 4 to 12) levels of CREB3L1 expression. We observed that all tumors resistant to doxorubicin-based chemotherapy fell in the group with lower expression of CREB3L1 (Fig. 3c). As a result, only 50% of the tumors in this group responded to the chemotherapy (Fig. 3c). In contrast, all tumors with higher expression of CREB3L1 were responsive to the chemotherapy (Fig. 3c).

## Discussion

We have previously reported that doxorubicin inhibits proliferation of tumor cells through proteolytic activation of CREB3L1, and CREB3L1 expression determines the sensitivity of tumors to doxorubicin in various *in vitro* and *in vivo* experimental systems [9, 12]. The current study demonstrates the clinical importance of the finding by establishing CREB3L1 expression as a potential biomarker capable of identifying TNBC patients who are likely to benefit from doxorubicin-based chemotherapy. We show that tumors with higher levels of CREB3L1 expression (IRS value ≥ 4) all responded to doxorubicin-based chemotherapy. Thus, applying such chemotherapy only to patients with higher levels of CREB3L1 expression should markedly increase the response rate of the treatment. We also observe that all tumors resistant to doxorubicin-based chemotherapy had lower levels of CREB3L1 expression (IRS value ≤ 3). However, some tumors with lower levels of CREB3L1 expression did respond to the chemotherapy. The most likely explanation for this observation is that TNBC patients are not treated with doxorubicin alone, and these tumors may be sensitive to other drugs included in the chemotherapy regimens such as Cytoxan and Taxol. If this is the case, then for tumors with lower levels of CREB3L1 expression, replacing doxorubicin with other chemotherapeutic reagents could prevent unnecessary cardiac toxicity associated with doxorubicin [6] without affecting the response rate of the treatment.

The current study, in which only 18 samples were analyzed, was originally designed as a pilot study to estimate the sample size required to reach statistical significance. Surprisingly, our small sample number was sufficient for us to draw the conclusion that CREB3L1 expression levels in tumors responsive to doxorubicin-based chemotherapy were higher than those resistant to the treatment. This is because the difference in CREB3L1 expression levels between the two groups of the tumors was so large (~ 8 fold) that even with such a limited sample size, statistical significance was achieved (*p* = 0.0005) though a simple student t-test. More importantly, power analysis, which has been established as the standard for sample size estimation in clinical studies [16], demonstrated that our statistic power (99.8%) was much higher than the value (80%) considered statistically significant in most clinical studies. Despite the small sample size, our sample selection was not skewed in terms of response of the tumors to chemotherapy, as the percentage of cases completely responsive or resistant to the therapy in our samples was similar to that in clinical trials involving hundreds of patients [3, 17]. Thus, adding more samples to this retrospect study will not further improve statistical significance in a clinically meaningful manner. Instead, this study should serve as a base for future prospective studies to firmly establish CREB3L1 as a clinical biomarker to identify TNBC patients who are most likely to respond to doxorubicin-based chemotherapy.

The current study reveals that CREB3L1 expression varies widely among different TNBC tumors. About 50% of the tumors we examined had low levels of CREB3L1 expression (IRS value ≤ 3). Previous reports have indicated that expression of CREB3L1 is frequently epigenetically silenced through DNA methylation in various cancers including breast cancer [18–21]. We have previously reported that azacitidine, a drug that inhibits DNA methylation [22], relieved epigenetic silencing of CREB3L1 [18]. In cultured MCF-7 cells in which CREB3L1 expression is barely detectable [9], azacitidine markedly increase the sensitivity of the cells to doxorubicin [23]. It will be interesting to determine whether addition of azacitidine to doxorubicin-based chemotherapy regimens may improve the treatment outcomes for TNBC with lower levels of CREB3L1 expression.

## Conclusion

The current study demonstrates that TNBC tumors with higher expression of CREB3L1 have a higher response rate to doxorubicin-based chemotherapy compared to those with lower expression of the protein. These results suggest that CREB3L1 expression level may serve as a biomarker to identify TNBC patients who are more likely to benefit from doxorubicin-based chemotherapy.

## Abbreviations

CREB3L1: Cyclic adenosine monophosphate response element binding protein 3 like-1
IHC: Immunohistochemistry
IRS: ImmunoReactive score
RCB: Residual cancer burden
TNBC: Triple negative breast cancer
